# Is greater public transport use associated with higher levels of physical activity in a regional setting? Findings from a pilot study

**DOI:** 10.1186/s40814-021-00951-8

**Published:** 2021-12-10

**Authors:** Bruna S. Ragaini, Melanie J. Sharman, Anna Lyth, Kim A. Jose, Leigh Blizzard, Corey Peterson, Fay H. Johnston, Andrew Palmer, Julie Williams, Elaine A. Marshall, Megan Morse, Verity J. Cleland

**Affiliations:** 1 Menzies Institute for Medical Research, University of Tasmania, 17 Liverpool St, Hobart, Tasmania 7000 Australia; 2 RED Sustainability Consultants, 54 Sandy Bay Road, Hobart, Tasmania 7004 Australia; 3 Infrastructure Services and Development, University of Tasmania, 20 College Road, Hobart, Tasmania 7001 Australia; 4 Department of Health, Tasmanian Government, GPO Box 125, Hobart, Tasmania 7001 Australia; 5 Metro Tasmania, PO Box 61, Moonah, Tasmania 7009 Australia

**Keywords:** Walking, Physical activity, Public transport, Active travel, Public health, Public policy

## Abstract

**Background:**

Public transport users often accumulate more physical activity than motor vehicle users, but most studies have been conducted in large metropolitan areas with multiple public transport options with limited knowledge of the relationship in regional and rural areas. In a regional city, this pilot study aimed to (1) test the feasibility of preliminary hypotheses to inform future research, (2) test the utility of survey items, and (3) establish stakeholder engagement.

**Methods:**

Data were collected via a cross-sectional online survey of 743 Tasmanian adults. Physical activity outcomes were walking (min/week), total moderate- to vigorous-intensity physical activity (min/week) and attainment of physical activity guidelines (yes/no). Transport variables were frequency of public and private transport use per week. Truncated and log binomial regression examined associations between public/private transport use and physical activity.

**Results:**

Neither frequency of public nor private transport use was associated with minutes of walking (public transport: *B* − 24.4, 95% CI: − 110.7, 61.9; private transport: *B* − 1.1, 95% CI: − 72.4, 70.1), minutes of total physical activity (public transport: *B* − 90.8, 95% CI: − 310.0, 128.5; private transport: *B* 0.4, 95% CI: − 134.0, 134.9) or not meeting physical activity guidelines (public transport: RR 1.02, 95%CI: 0.95, 1.09; private transport: RR 1.02, 95%CI: 0.96, 1.08).

**Conclusions:**

The hypothesis that public transport users would be more physically active than private transport users was not supported in this pilot study. Stakeholders were engaged and involved in various phases of the research including development of research questions, participant recruitment, and interpretation of findings. Further studies using representative samples and refined measures are warranted to confirm or refute findings.

**Supplementary Information:**

The online version contains supplementary material available at 10.1186/s40814-021-00951-8.

## Key messages regarding feasibility

- The relationship between physical activity and public transport use in regional and rural areas is not clear.
- A relationship between physical activity and public transport use in this regional area was not supported, but useful information to inform future studies was collected, and stakeholder partnerships were strengthened.
- Future studies using representative samples and refined measures are warranted to confirm or refute findings.

## Background

Despite the well-documented role that regular participation in physical activity (PA) plays in reducing the public health burden of disease, the prevalence of insufficient PA is still sizeable in Australia and internationally. An insufficiently active population is at greater risk of developing non-communicable diseases such as coronary heart disease, dementia, diabetes, stroke, depression and different types of cancer [1–5]. Maintaining sufficient levels of PA are associated with weight control, improved muscular and cardiorespiratory fitness and improved bone and functional health [6]. The estimated total costs of inactivity were INT$567.5 billion globally and AUD$805 million in Australia in 2013 [7]. Nonetheless, only 69% of adults (15 years or older) worldwide meet the recommendation of at least 150 min of moderate-intensity PA per week [8]. In Australia, 48% of adults (18–64 years old) are classed as sufficiently active [9]. Efforts to increase levels of PA in the population through the promotion of leisure-time activity have failed to attain the desired outcome, so there is a need to target other domains of PA [10].

Incidental PA—or unstructured activity that is accumulated during the day—has the potential to offer significant benefits to public health. Engagement in active transport—such as walking and cycling—offers one way of accumulating incidental PA. A systematic review found that public transport users accumulate 8–15 additional minutes of total PA per day, making them 3.5 times more likely to meet PA guidelines compared with motor vehicle users [11]. However, whether public transport users are more physically active than private transport users in non-metropolitan areas remains unknown, as most studies consider transport in the context of densely populated metropolitan areas with an abundance of public transport options running at high frequencies.

Neighbourhood characteristics, such as accessibility to public transport and destination proximity [12–21], as well as the general structure and design of the public spaces of a city [22, 23], have been identified as significant factors in encouraging higher levels of engagement with active transport. Transport systems in particular have been identified in numerous international [24] and national [25] frameworks as an aspect of the built environment that can have a critical impact on health and behaviour.

The Australian island state of Tasmania is a regional area where buses are the only mode of public transport, and metropolitan services are predominantly offered by a single provider. Only 3% of Tasmanian adults use public transport as their primary mode of transport on their journey to work [26], and it is estimated that it comprises only 4% of the primary trips for all purposes in the greater capital city region of Hobart [27]. In 2014–2015, Tasmania had the lowest proportion of adults engaged in sufficient amounts of PA in the country at 43% [28]. This low engagement with both public transport and PA indicates a potential opportunity to simultaneously increase levels of PA and public transport use for health, environmental, social, and economic benefit. However, prior to intervention, the relationship between PA and public transport in non-metropolitan areas requires investigation and confirmation. The aims of this pilot study were (1) to test the preliminary hypothesis that greater public transport use was associated with higher PA levels among a sample of adults living in a regional area, (2) test the utility of survey items, and (3) establish stakeholder engagement.

## Methods

Data were from the cross-sectional 2017 Tasmanian Travel and Physical Activity Survey (TAPAS) pilot observational study. TAPAS was conducted to generate information about travel behaviour and physical activity in a regional area, generate information for sample size estimations for future work, engage with stakeholders (public transport provider, local government, state government) and establish whether a larger observational study was warranted. Ethical approval was received on 10 February 2017 (H16327), and participants were required to review an information sheet and provide informed consent. We used the STROBE Statement to guide reporting (Additional File 1).

### Stakeholder engagement

Stakeholder engagement encompassed principles outlined in the VicHealth Stakeholder Engagement Framework (meaningful, open, inclusive, respectful and collaborative) [29]. We engaged with a public transport provider, the local government peak body, a state government health department, a state government planning department, a local council, a university sustainability department, and a sustainable transport consultant. Connections with stakeholders occurred by consulting (seeking advice on and impact of proposed work through meetings and informal discussions), collaborating (partnering for development and delivery of jointly agreed work through this research), and empowering (supporting stakeholders in actions to build a healthier Tasmania).

### Study population

Tasmania has a population approaching 520,000 people [30]; only the Northern Territory (247,000) and Australian Capital Territory (431,800) are smaller. The largest Australian state, New South Wales, has a population of 8,176,400. Approximately 43% of Tasmania’s population resides in and around the Greater Hobart Region [31], making it one of Australia’s least populated cities [32]; comparatively, the capital cites of Darwin (Northern Territory) and Canberra (Australian Capital Territory) have around 147,000 and 431,00 residents, respectively, while the capital city of New South Wales (Sydney) has more than 5 million residents. While 72% of Australians live in ‘Major Cities’, there are no areas classified as Major Cities in Tasmania.

**Fig. 1:**
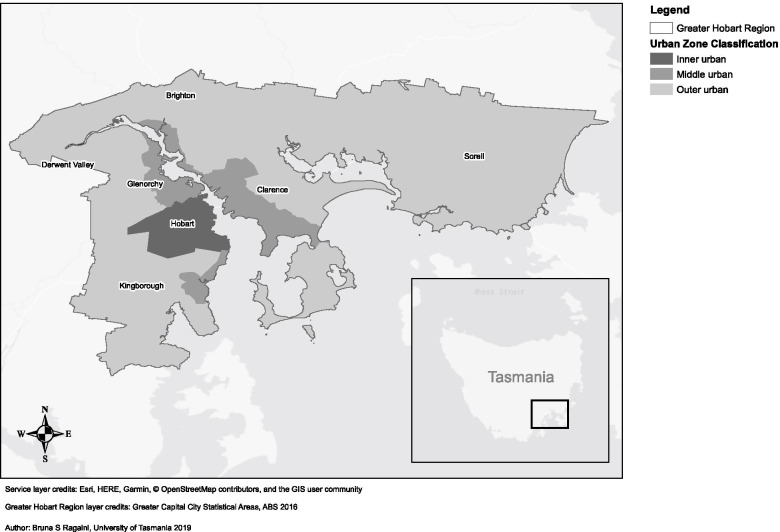
Urban zone classification in the Greater Hobart Region, Tasmania, Australia

The Greater Hobart Region includes the capital city of Hobart, as well as surrounding local government areas (LGAs) (Fig. 1). Hobart is classified as an ‘Inner Regional’ area using the Australian Statistical Geography Standard [33], and is closest in size to the Queensland cities of Townsville (183,000) and Cairns (155,000), both considered ‘Outer Regional’ areas. The region has a low-density settlement pattern, with a large proportion of single-detached dwellings found in residential areas. Ninety two percent of all jobs in Southern Tasmania are within the Greater Hobart Region; of these, 47% are housed in the Hobart City Council LGA, followed by 31% in the satellite LGAs of Glenorchy and Clarence. Despite a recent trend towards multiple commercial centres within the region, the public transportation system remains largely radial [34]. Low residential density (and the resulting limited public transport options), housing affordability and limited local employment opportunities have driven the population to become heavily reliant on private motor vehicles [35].

### Recruitment methods

Eligible participants were adults aged 18+ years living in Tasmania in March and April 2017. Convenience recruitment methods included dissemination of promotional materials in key locations and via professional networks and organisations, social media, and traditional media. Participants opted into a draw to win one of five AUD$100 vouchers.

### Procedure and sample size

As this was a pilot study, we did not conduct sample size calculations. From a total of 1355 adults who participated in the TAPAS study, 612 were excluded from this analysis. Among these, 264 failed to answer all mandatory questions (including key demographic measures), and 147 contained missing data on PA variables due to a systematic error in the survey design. An investigation of the potential bias in demographic characteristics of the group with missing data compared with the group with complete data revealed no statistically significant differences at the 95% confidence level between the two groups (Additional File 2). This result guided the decision to exclude these participants from the analysis. In addition, only those living in the relatively well-serviced Greater Hobart Region who used public and/or private transport during the observed week were included in this study, resulting in a sample size of 743.

### Measures

Self-reported duration and frequency of walking and vigorous- and moderate-intensity PA were collected via the short form of the International Physical Activity Questionnaire—Short Form (IPAQ-SF) [36]. The IPAQ standardised scoring protocol [37] was used to derive three variables: minutes of weekly walking, minutes of weekly total PA and a binary variable to indicate whether the Australian National PA Guidelines [38] had been met. Walking was selected as an outcome because conceptually it would be the physical activity behaviour most likely to be influenced by mode of transport, especially as there is no capacity to store bicycles on buses in Tasmania. Total PA was calculated by adding minutes of walking and vigorous- and moderate-intensity PA. Meeting PA guidelines was defined as *no* (less than 150 min of total PA or 75 min of vigorous-intensity PA) or *yes* (at least 150 min of total PA or 75 min of vigorous-intensity PA).

Public and private transport use were derived from a past week trip recall survey that collected data on the purpose, mode of transport and duration of each trip for each day of the week. Public and private transport use was defined as the frequency of use in the 1-week period captured by the survey. Participants were categorised as public transport users and nonusers for each day of the week (yes = 1, no = 0), resulting in a measure ranging from 0 to 7 days of public or private transport use per week.

Sociodemographic variables included age (18–24, 25–34, 35–44, 45–54 or 55+ years); highest level of education (low, medium or high); employment status (employed full-time, employed part-time or not in the workforce and other); household composition (family with children < 18 years old living at home, couple without children < 18 years old living at home, group household or other); self-reported health (excellent, very good, good or fair/poor), gender (man, woman or other); student (studying full-time or not studying full-time), language spoken at home (English or other) and current injury, illness, disability restricting PA (yes or no). Other measures included walking distance from home to the nearest bus stop (5 min or less, 6 to 10 min, more than 10 min), access to a motor vehicle (yes or no) and whether physical activity in the last week was the same or different than usual (same as usual or different than usual). A variable derived from participants’ postcodes indicated residence in inner, middle or outer Greater Hobart urban zones. Inner urban was defined as all suburbs within the City of Hobart; middle urban refers to the well-serviced broader urban area [39]; and outer urban refers to remaining suburbs within the Greater Hobart Region where public transport services are less frequent and accessible (Fig. 1). This urban zone classification was generated to investigate well-established geographic differences in transport behaviour [40–42].

### Analysis

Descriptive statistics (medians and interquartile ranges for continuous data and numbers and proportions for categorical data) were used to characterise the sample. A confounder analysis investigated which variables were associated with both the outcome and predictor variables (Additional File 3). Only correlates that satisfied the forward stepwise selection criteria were included as confounders in each of the adjusted models. The associations between continuous outcomes and predictors and the categorical and ordinal variables were tested using the Kruskal–Wallis test (or Mann–Whitney U test for binary variables) and Spearman correlation, respectively. The associations between the dichotomous outcome and the categorical and ordinal variables were tested using chi-squared (or Fisher’s Exact test) and Kruskal–Wallis tests (or Mann–Whitney U test), respectively. A conservative alpha was set at 0.2.

The use of linear regression to investigate the association between frequency of public and private transport use and weekly minutes of walking and total PA was inappropriate due to the nonnormal distribution of the PA variables and the presence of zeros in the data that would have generated missing values if the outcomes were log transformed. PA variables were truncated as per IPAQ scoring protocols, and their distribution lent themselves to truncated regression models.

Unadjusted and adjusted log binomial regression models were built to estimate the relationship between frequency of public and private transport use per week and the risk of participants not meeting PA guidelines. Household composition and employment status were collapsed into dichotomous variables (i.e., family with children vs. couples without children/group household/lone person, working full- or part-time hours vs. not in the labour force) after the multivariable log binomial models failed to converge. These categories were chosen after descriptive statistics indicated similar distributions when cross-referenced with PA outcomes.

All analyses were conducted using Stata SE 15.0 (StataCorp, TX, USA). Each regression model was subjected to analysis of model fit performed using a forward stepwise selection method. Nested models were compared, and confounding variables were selected for inclusion in the model based on a 10% change threshold to the coefficient of the exposure variable [43].

## Results

Sample characteristics of the 743 participants can be found in Table 1. Compared with the broader Greater Hobart population, the following groups were overrepresented in the survey: women (67% in this study vs. 52% of the broader population), people aged 18–54 (85% vs. 60%), those working part- or full-time (78% vs. 56%), full-time students (23% vs. 6%), those with a university qualification (57% vs. 26%) and those who speak English at home (97% vs. 92%) [44].

**Table 1:** Sample characteristics (*n* = 743)

	Sample % (***n***)
**Gender**	
Man	32.4 (241)
Woman	66.9 (497)
Other^*^	0.7 (5)
**Age**	
18–24	17.4 (129)
25–34	24.1 (179)
35–44	23.0 (171)
45–54	20.6 (153)
55+	14.9 (111)
**Employment status**	
Working full-time hours	46.0 (342)
Working part-time hours	31.6 (235)
Not in the labour force	21.5 (160)
Other^†^	0.8 (6)
**Student**	
Studying full-time	23.3 (173)
Not studying full-time	76.7 (570)
**Highest education level**^‡^	
Low	20.3 (151)
Medium	23.0 (171)
High	56.7 (421)
**Household composition**	
Family with children < 18 years old living at home	33.7 (250)
Couple without children < 18 years old living at home	24.4 (181)
Group household (adults living together)	20.5 (152)
Lone person	19.0 (141)
Other^§^	2.6 (19)
**Language spoken at home**	
English	97.3 (723)
Other	2.7 (20)
**General health**	
Excellent	19.7 (150)
Very good	39.4 (293)
Good	29.6 (218)
Fair/poor	11.3 (82)
**Current injury, illness, disability restricting physical activity**	
Yes	12.9 (96)
No	87.1 (647)
**Urban zone classification**	
Inner urban	45.1 (335)
Middle urban	46.0 (342)
Outer urban	8.9 (66)
**Walking distance from home to bus stop**	
5 min or less	64.3 (478)
6 to 10 min	21.7 (161)
More than 10 minutes	14.0 (104)
**Has access to a motor vehicle/cycle**	
Yes	77.9 (579)
No	22.1 (164)
**Physical activity last week the same of different than usual**	
The same as usual	85.1 (632)
Different than usual	14.9 (111)
**Physical activity guidelines**	
Meets guidelines	79.0 (587)
Does not meet guidelines	21.0 (156)
	**Median (**^||^**)**
**Minutes of physical activity/week**	
Walking	175 (80–280)
Total physical activity	310 (170–530)
**Frequency of transport use/week**	
Public transport	0 (0–2)
Private transport	5 (2–7)

* Includes ‘transgender’, ‘prefer not to disclose’, ‘gender fluid’ and ‘non-binary’; † includes volunteers and unclear responses; ‡ Low = year 12 or less, Medium = trade/apprenticeship or certificate/diploma, High = university qualification, § includes large families, multigenerational households, visitor and unclear responses; || 25th and 75th quartiles

Sixty-one percent of survey participants travelled only by motor vehicle, 11% travelled only by public transport, and 28% used mixed modes of transport. Median frequency of motor vehicle use was 5 days/week, and median frequency of public transport use was 0 days/week. Median time spent in PA was 175 min per week for walking and 310 min/week for total PA, and 79% of participants met Australian PA guidelines.

The effects of frequency of public and private transport use on time spent walking and doing total PA were negligible, with no statistically significant relationship found in unadjusted or adjusted models (Table 2). Frequency of public and private transport use also had no significant association with the likelihood of meeting PA guidelines in unadjusted or adjusted models.

**Table 2:** Associations between transport mode and physical activity (PA) outcomes

	Public transport		Private transport	
	Unadjusted	Adjusted	Unadjusted	Adjusted
Walking min/week, *B* (95% CI)*	29.4 (− 76.5, 135.4)	− 24.4 (− 110.7, 61.9)^a^	− 60.0 (− 155.4, 35.4)	− 1.1 (− 72.4, 70.1)^a^
Total PA min/week, *B* (95% CI)*	− 74.2 (− 314.9, 166.4)	− 90.8 (− 310.0, 128.5)^b^	− 65.0 (− 246.7, 116.8)	0.4 (− 134.0, 134.9)^c^
Not meeting PA guidelines, RR (95% CI)^†^	1.05 (0.98, 1.13)	1.02 (0.95, 1.09)^d^	1.01 (0.96, 1.07)	1.02 (0.96, 1.08)^e^

* Coefficient and 95% confidence intervals estimated using truncated regression; † risk ratio and 95% confidence intervals estimated using log binomial regression; ‡ includes ‘couple without children < 18 years old living at home’, ‘group household (adults living together)’ and ‘lone person’

^a^ Adjusted for household composition, access to a motor vehicle, urban zone classification

^b^ Adjusted for age, education, household composition, urban zone classification

^c^ Adjusted for gender, age, education, household composition, urban zone classification

^d^ Adjusted for employment status, urban zone classification

^e^ Adjusted for gender, employment status, urban zone classification

## Discussion

The primary aim of this study was to determine whether higher frequency of public transport use was associated with more PA in a regional city. We found that neither frequency of public nor private transport use had a significant relationship with PA in this sample. This finding contrasts with existing literature where public transport users are more physically active than private transport users [11]. A likely possible explanation for these discrepant findings is that previous studies have predominantly been conducted in large metropolitan centres with more than one public transport option, where a single journey may consist of multimodal trips. Hobart, on the other hand, is a regional city with a single type of public transport available (buses) predominantly serviced by a single provider (Metro Tasmania).

Previous PA and transport studies have shown that ‘place’ matters and that urban design may be associated with engagement in both PA and public transport use [12, 14, 16, 17, 20–22]. For instance, distance to a bus stop and housing density have been found to be significantly associated with regular walking for transport [12, 14]. Urban sprawl is an issue in the low-density Greater Hobart Region and walking to the bus stop is not always viable for those living on the urban fringe, semi-rural environments, or small towns with more limited access to bus routes. To address this accessibility issue, there has been a focus on “park and ride” facilities to encourage public transport use for those living outside the Hobart LGA, for instance, in the LGA of Kingborough [45]. However, those driving to the bus stop are unlikely to be benefiting from the active components often associated with public transport use. While the relationship between PA and public and private transport use may therefore differ according to whether people live in the inner, middle or outer Greater Hobart urban zones, we found no evidence of an interaction between public and private transport use and urban zone in this sample (data not shown).

Bus stop accessibility and parking zones in Hobart’s Central Business District (CBD) may be another explanation for why public transport users may not be accumulating more PA than private transport users in this regional setting. The availability of all-day on-street motor vehicle parking in the CBD is decreasing. Consequently, some drivers *may* choose to park for free or at a low cost in inner suburban areas and walk the remainder of the way [46]. On the other hand, an abundance of bus stops within the CBD possibly translates into shorter walking trips for public transport users. While private transport users may be taking longer walking trips during their journeys, public transport users may be taking multiple shorter walking trips (e.g., public transport users walk to the bus stop then walk to the destination, while motor vehicle drivers only walk to the destination). Future research should consider investigating how urban sprawl, parking zones and public transport systems in nonmetropolitan areas encourage or discourage higher engagement with PA through public transport use. Further, strategies to increase PA through public transport use that target the inner and middle urban zones may be more likely to succeed, due to destination proximity, urban design and public transport accessibility.

A second aim was to establish the utility of the measures used. Substantial amounts of missing demographic data (*n* = 264) suggest improvements to our survey tool are required. Other issues with the measures used that were identified include the IPAQ-S potentially not being sensitive enough to detect associations with transport mode frequency, despite broad acceptance as a valid measure of PA [37]. Studies that have found associations between public transport use and increased PA have used a combination of objective measures (i.e., pedometers and/or accelerometers) and self-report trip diaries or diary logs. We identified only two other studies [47, 48] that used the IPAQ (both long and short forms), and neither found a significant association between public transport and quantity of PA, although one found that transport mode had an effect on PA participation [47]. Further, some studies have exclusively examined transport-related PA, while the current study focused on walking for all purposes and total PA [13, 49, 50]. Further refinement of measures for future studies of transport behaviour and PA is required.

This study also aimed to establish stakeholder engagement. One stakeholder group that we consulted with did not engage further with the project, largely due to a lack of capacity and management support. The remaining stakeholders were involved in establishing research questions, supporting participant recruitment, and interpreting findings, ensuring local relevance and establishing a direct pipeline for research translation. The strength of engagement was further demonstrated through a subsequent successful funding application to Australia’s National Health and Medical Research Council Partnership Project scheme, where three of these partners (public transport provider, local government peak body, state government health department) committed substantial in-kind and/or cash contributions to advance this work. It is widely acknowledged that intersectoral action is required to support uptake of active and public transport [24, 51, 52]. A novel aspect of this partnership was the bringing together of stakeholders from research, policy, and practice across the health and public transport sectors.

If findings from this pilot study were replicated in a representative sample using refined measures, it would suggest that strategies solely based on the promotion of public transport use in nonmetropolitan areas to increase PA may have limited success without support from other interventions targeting individual (e.g., behaviour change programs), social (e.g., cultural norms) and/or environmental (e.g., urban form) factors. This poses challenges to current transport- and health-related policies and strategies that aim to promote more active living by encouraging a change from private to public transport in the Greater Hobart Region [22, 53]. The Tasmanian Government has promoted walking to or from a more distant bus stop for PA gain [54], but the uptake of this message is limited [55]. Further research is needed to explore these issues and possible intervention strategies that may positively impact on both physical activity behaviour and healthy transport options.

### Strengths and limitations

This pilot study had limitations. First, as a pilot study, generalisation of the findings to the wider population is limited because the sample was not drawn at random. Although the sample showed heterogeneity among the participants in key demographic characteristics, PA profiles and transport behaviours, it differed from the broader population in several ways (as described in the Results section). The online survey measured transport use related to the past week, but these may not reflect participants’ usual behaviour. The short form of the IPAQ does not differentiate between leisure time and transport-related PA which impeded the investigation of the direct association between transport mode on incidental PA. The IPAQ overestimates physical activity [56]. This overestimation is problematic at a population level (e.g., for prevalence estimates based on absolute values), but is not of concern in this study where the intention was to make between-group comparisons.

There were strengths to this pilot study. It is the first study to test hypotheses around public transport use and PA in a regional setting, contributing new knowledge to the evidence base. The large sample enabled multivariable analyses that considered a broad range of potential confounding factors. Further, it used a broadly accepted measure of PA that, despite some validity concerns [56], offers reliable and results comparable with other studies. It also provided an opportunity to engage closely with stakeholders, including a public transport provider, local government peak body, and state government health department, to codesign research questions, support recruitment and interpret findings.

## Conclusion

This study investigated the associations between public and private transport use and PA in the regional city of Hobart, Australia. It found that neither frequency of public nor private transport use was associated with PA in this population. The findings require confirmation in representative samples, both in Tasmania and in other regional areas with similar population size, density and topography, and the use of objective measures of transport and PA behaviour.

## Supplementary Information

**Additional file 1: Table S1**. STROBE Statement checklist.

**Additional file 2: Table S2**. Examination of associations* between missing values and demographic characteristics in the Transport and Physical Activity Study (n=1,091).

**Additional file 3: Table S3**. Results of the confounder analysis examining the associations between sociodemographic variables and frequency of public and private transport use* and physical activity outcomes† (n=743).

## Data Availability

The datasets used and/or analysed during the current study are available from the corresponding author on reasonable request.

## Abbreviation

PA: Physical activity

## References

[1] Australian Institute of Health and Welfare. Impact of physical inactivity as a risk factor for chronic conditions: Australian Burden of Disease Study. Cat no. BOD 16 2017 [Available from: https://www.aihw.gov.au/getmedia/df392a65-8cf3-4c09-a494-4498ede2c662/aihw-bod-16.pdf.aspx?inline=true.

[2] Lautenschlager NT, Cox KL, Flicker L, Foster JK, van Bockxmeer FM, Xiao J (2008). Effect of physical activity on cognitive function in older adults at risk for Alzheimer disease: a randomized trial. JAMA..

[3] Kyu HH, Bachman VF, Alexander LT, Mumford JE, Afshin A, Estep K (2016). Physical activity and risk of breast cancer, colon cancer, diabetes, ischemic heart disease, and ischemic stroke events: systematic review and dose-response meta-analysis for the Global Burden of Disease Study 2013. Br Med J.

[4] Rebar AL, Stanton R, Geard D, Short C, Duncan MJ, Vandelanotte C (2015). A meta-meta-analysis of the effect of physical activity on depression and anxiety in non-clinical adult populations. Health Psychol Rev.

[5] Brister M (2018). Systematic review on the role of exercise in cardiovascular disease. Doctoral Dissertation.

[6] World Health Organization. Physical activity. 2018 [Available from: http://www.who.int/mediacentre/factsheets/fs385/en/

[7] Ding D, Lawson KD, Kolbe-Alexander TL, Finkelstein EA, Katzmarzyk PT, Van Mechelen W (2016). The economic burden of physical inactivity: a global analysis of major non-communicable diseases. Lancet.

[8] Hallal PC, Andersen LB, Bull FC, Guthold R, Haskell W, Ekelund U (2012). Global physical activity levels: surveillance progress, pitfalls, and prospects. Lancet.

[9] Australian Institute of Health and Welfare. Physical activity across the life stages. Cat no. PHE 225 2018 [Available from: https://www.aihw.gov.au/getmedia/c249ef97-e219-44df-a8bd-f5e50d04064c/aihw-phe-225.pdf.aspx?inline=true.

[10] Bauman AE, Reis RS, Sallis JF, Wells JC, Loos RJ, Martin BW (2012). Correlates of physical activity: why are some people physically active and others not?. Lancet.

[11] Rissel C, Curac N, Greenaway M, Bauman A (2012). Physical activity associated with public transport use—a review and modelling of potential benefits. Int J Environ Res Public Health.

[12] McCormack GR, Giles-Corti B, Bulsara M (2008). The relationship between destination proximity, destination mix and physical activity behaviors. Prev Med.

[13] Morabia A, Amstislavski PN, Mirer FE, Amstislavski TM, Eisl H, Wolff MS (2009). Air pollution and activity during transportation by car, subway, and walking. Am J Prev Med.

[14] Coogan PF, White LF, Adler TJ, Hathaway KM, Palmer JR, Rosenberg L (2009). Prospective study of urban form and physical activity in the Black Women's Health Study. Am J Epidemiol.

[15] Brown BB, Werner CM (2007). A new rail stop: tracking moderate physical activity bouts and ridership. Am J Prev Med.

[16] Cleland VJ, Timperio A, Crawford D (2008). Are perceptions of the physical and social environment associated with mothers' walking for leisure and for transport? A longitudinal study. Prev Med.

[17] Frank LD, Greenwald MJ, Winkelman S, Chapman J, Kavage S (2010). Carbonless footprints: promoting health and climate stabilization through active transportation. Prev Med.

[18] Lachapelle U, Frank LD (2009). Transit and health: mode of transport, employer-sponsored public transit pass programs, and physical activity. J Public Health Policy.

[19] Li F, Harmer PA, Cardinal BJ, Bosworth M, Acock A, Johnson-Shelton D (2008). Built environment, adiposity, and physical activity in adults aged 50–75. Am J Prev Med.

[20] Liao Y, Harada K, Shibata A, Ishii K, Oka K, Nakamura Y (2011). Perceived environmental factors associated with physical activity among normal-weight and overweight Japanese men. Int J Environ Res Public Health.

[21] McConville ME, Rodriguez DA, Clifton K, Cho G, Fleischhacker S (2011). Disaggregate land uses and walking. Am J Prev Med.

[22] National Heart Foundation of Australia. Healthy by Design®. A guide to planning and designing environments for active living in Tasmania 2009 [Available from: https://www.heartfoundation.org.au/images/uploads/publications/Healthy-by-Design-Tasmania.pdf.

[23] Christiansen LB, Cerin E, Badland H, Kerr J, Davey R, Troelsen J (2016). International comparisons of the associations between objective measures of the built environment and transport-related walking and cycling: IPEN adult study. J Transp Health.

[24] World Health Organization (2018). Global action plan on physical activity 2018–2030: more active people for a healthier world.

[25] National Heart Foundation of Australia. Blueprint for an active Australia 2019 [Available from: https://www.heartfoundation.org.au/images/uploads/publications/Blueprint/Blueprint_For_An_Active_Australia_Third_Edition.pdf.

[26] Department of Infrastructure, Energy and Resources. Journey to work data analysis: an analysis of 2011 Australian Bureau of Statistics Census data relating to journey to work pattern: Tasmanian government; Tasmanian Government. 2011 [Available from: https://www.stategrowth.tas.gov.au/__data/assets/pdf_file/0005/88610/Journey_to_Work_Report_2011_Census_Analysis.pdf.

[27] Infrastructure Strategy Division, Department of Infrastructure, Energy and Resources. The Greater Hobart Household Travel Survey: summary of analysis and key findings: Tasmanian Government; Tasmanian Government. 2010 [Available from: https://www.transport.tas.gov.au/__data/assets/pdf_file/0003/109731/Household_Travel_Survey_Summary_-_Final.pdf.

[28] Australian Bureau of Statistics. National Health Survey: First Results, 2014-15. Cat no. 4364.0.55.001; 2015.

[29] VicHealth. Stakeholder Engagement Framework 2018-23. Victoria, Australia: Victorian Health Promotion Foundation; 2018.

[30] Australian Bureau of Statistics. Census of population and housing: reflecting Australia. stories from the census, 2016 - snapshot of Australia. Cat no. 2071.0; 2016.

[31] Australian Bureau of Statistics. Regional population growth, Australia, 2016. Cat no. 3218.0; 2017.

[32] Australian Bureau of Statistics. Regional population growth, Australia, 2016-17. Cat no. 3218.0; 2018.

[33] Australian Bureau of Statistics (2011). Australian Statistical Geography Standard (ASGS): volume 5 - remoteness structure.

[34] Tasmanian Planning Commission. Southern Tasmania Regional Land Use Strategy 2010-2035 Southern Tasmanian Councils Authority. 2018 [Available from: https://www.planning.tas.gov.au/__data/assets/pdf_file/0004/332986/Southern_Tasmania_Regional_Land_Use_Strategy_-_Amended_Effective_9_May_2018.pdf.

[35] Department of Infrastructure, Energy and Resources. Congestion in Greater Hobart: response to issues Tasmanian Government. 2011 [Available from: https://www.transport.tas.gov.au/__data/assets/pdf_file/0016/110644/Greater_Hobart_Congestion_full_report.pdf.

[36] IPAQ Research Committee. Guidelines for data processing and analysis of the International Physical Activity Questionnaire (IPAQ)-short and long forms 2005 [Available from: http://www.ipaq.ki.se/scoring.pdf.

[37] Craig CL, Marshall AL, Sjorstrom M, Bauman AE, Booth ML, Ainsworth BE (2003). International physical activity questionnaire: 12-country reliability and validity. Med Sci Sports Exerc.

[38] Department of Health. Australia's physical activity and sedentary behaviour guidelines for adults (18-64 years) Australian government. 2014 [Available from: https://www1.health.gov.au/internet/main/publishing.nsf/Content/health-pubhlth-strateg-phys-act-guidelines.

[39] Department of State Growth. Urban area maps Tasmanian Government. 2019 [Available from: https://www.transport.tas.gov.au/passenger/passengers/student_travel/urban_area_maps

[40] Hanson S, Giuliano G. The geography of urban transportation: Guilford Press; 2004.

[41] Ewing R, Cervero R (2001). Travel and the built environment: a synthesis. Transport Res Record J Transport Res Board.

[42] Geurs KT, Van Wee B (2004). Accessibility evaluation of land-use and transport strategies: review and research directions. J Transp Geogr.

[43] Greenland S (1989). Modeling and variable selection in epidemiologic analysis. Am J Public Health.

[44] Australian Bureau of Statistics. 2016 Census of Population and Housing. TableBuilder. Findings based on use of ABS TableBuilder data 2016 [

[45] Kingborough Council. Kingborough Integrated Transport Strategy 2010 [Available from: https://www.kingborough.tas.gov.au/wp-content/uploads/2017/05/Kingborough-Integrated-Transport-Strategy-2010-r.pdf.

[46] Hobart City Council. Parking - a plan for the future 2013 2013 [Available from: https://www.hobartcity.com.au/Council/Strategies-and-plans/Parking-A-Plan-for-the-Future.

[47] Wener RE, Evans GW (2007). A morning stroll: levels of physical activity in car and mass transit commuting. Environ Behav.

[48] Shaw C, Keall M, Guiney H (2017). What modes of transport are associated with higher levels of physical activity? Cross-sectional study of New Zealand adults. J Transp Health.

[49] Besser LM, Dannenberg AL (2005). Walking to public transit: steps to help meet physical activity recommendations. Am J Prev Med.

[50] Evans AW, Addison JD (2009). Interactions between rail and road safety in Great Britain. Accid Anal Prev.

[51] National Heart Foundation of Australia (2019). Blueprint for an active Australia.

[52] Bellew B, Nau T, Smith B, Bauman A (2020). Getting Australia active III: a systems approach to physical activity for policy makers.

[53] Hobart City Council. City of Hobart Transport Strategy 2018-30 2018 [Available from: https://www.hobartcity.com.au/Council/Strategies-and-plans/City-of-Hobart-Transport-Strategy-2018-30.

[54] Department of Premier and Cabinet. Get Moving Tasmania Tasmanian Government. 2018 [Available from: http://www.getmoving.tas.gov.au/move_more_sit_less

[55] Ragaini BS, Sharman M, Lyth A, Jose K, Blizzard CL, Peterson C, et al. A mixed-methods study of the demographic and behavioural correlates of walking to a more distant bus stop. Transport Res Interdisciplinary Perspect. 2020;6.

[56] Lee PH, Macfarlane DJ, Lam TH, Stewart SM (2011). Validity of the International Physical Activity Questionnaire Short Form (IPAQ-SF): a systematic review. Int J Behav Nutr Phys Act.

