# Supplementary material for: Is greater public transport use associated with higher levels of physical activity in a regional setting? Findings from a pilot study
Source: Pilot Feasibility Stud. 2021 Dec 10;7:217. doi: 10.1186/s40814-021-00951-8 (PMC8662899; doi:10.1186/s40814-021-00951-8)
Supplement: Supplementary file 2 — Additional file 2: Table S2. Examination of associations* between missing values and demographic characteristics in the Transport and Physical Activity Study (n=1,091). [file 40814_2021_951_MOESM2_ESM.docx]

| **Variables** | **% Missing (n)** |
| --- | --- |
| **Gender, p** | 0.259 |
| Man | 14.9 (52) |
| Woman | 12.5 (91) |
| Other† | 33.3 (4) |
| **Age, p** | 0.368 |
| 18-24 | 16.4 (34) |
| 25-34 | 13.3 (34) |
| 35-44 | 15.0 (37) |
| 45-54 | 10.2 (21) |
| 55+ | 11.9 (21) |
| **Employment status, p** | 0.917 |
| Working full-time hours | 13.8 (67) |
| Working part-time hours | 13.2 (45) |
| Not in the labour force | 12.8 (33) |
| Other‡ | 22.2 (2) |
| **Student, p** | 0.193 |
| Studying full-time | 15.9 (41) |
| Not studying full-time | 12.7 (106) |
| **Highest education level**§**, p** | 0.959 |
| Low | 13.5 (33) |
| Medium | 14.0 (38) |
| High | 13.2 (76) |
| **Household composition, p** | 0.652 |
| Family with children <18yo living at home\|\| | 14.7 (52) |
| Couple without children <18yo living at home | 13.7 (39) |
| Group household (adults living together) | 13.7 (30) |
| Lone person | 10.9 (22) |
| Other¶ | 12.5 (4) |
| **Language spoken at home, p** | 0.297**^◊^** |
| English | 13.6 (145) |
| Other | 7.7 (2) |
| **General health, p** | 0.068 |
| Excellent | 16.9 (37) |
| Very good | 13.4 (56) |
| Good | 13.8 (46) |
| Fair/poor | 6.6 (8) |
| **Current injury, illness, disability restricting physical activity, p** | 0.713 |
| Yes | 12.5 (18) |
| No | 13.6 (129) |
| * Chi-square test unless otherwise indicated; † includes ‘transgender’, ‘prefer not to disclose’, ‘gender fluid’ and ‘non-binary’ (excluded from test statistic); ‡ includes volunteers and unclear responses (excluded from test statistic); § Low = Year 12 or less; Medium = Trade/apprenticeship or Certificate/Diploma; High = University qualification; \|\| includes ‘one parent family’; ¶ includes large families, multigenerational households, ‘visitor only’ and unclear responses (excluded from test statistic); **◊** Fisher’s exact test | |
