# Supplementary material for: Is greater public transport use associated with higher levels of physical activity in a regional setting? Findings from a pilot study
Source: Pilot Feasibility Stud. 2021 Dec 10;7:217. doi: 10.1186/s40814-021-00951-8 (PMC8662899; doi:10.1186/s40814-021-00951-8)
Supplement: Supplementary file 3 — Additional file 3: Table S3. Results of the confounder analysis examining the associations between sociodemographic variables and frequency of public and private transport use* and physical activity outcomes† (n=743). [file 40814_2021_951_MOESM3_ESM.docx]

|  | **Median (25^th^ and 75^th^ quartiles)** | | | | **% (n)** | |
| --- | --- | --- | --- | --- | --- | --- |
|  | **Public transport** | **Private transport** | **Walking min/week** | **Total physical activity min/week** | **Meets physical activity guidelines** | |
| **Gender, p**^*^ | 0.865 | **0.070** | 0.670 | **0.000** | **0.000**^†^ | |
| Man | 0 (0-2) | 4 (2-6) | 175 (75-280) | 370 (210-590) | 87.1 (210) | |
| Woman | 0 (0-2) | 5 (2-7) | 150 (80-280) | 290 (145-480) | 75.3 (374) | |
| **Age, p**‡ | **0.000** | **0.000** | 0.235 | **0.171** | 0.635^*^ | |
| 18-24 | 1 (0-4) | 2 (1-6) | 175 (90-245) | 310 (180-545) | 79.1 (102) | |
| 25-34 | 0 (0-2) | 5 (2-7) | 150 (80-245) | 270 (150-470) | 77.1 (138) | |
| 35-44 | 0 (0-2) | 5 (3-7) | 160 (80-280) | 320 (160-475) | 80.1 (137) | |
| 45-54 | 0 (0-1) | 5 (3-7) | 150 (75-300) | 330 (165-540) | 78.4 (120) | |
| 55+ | 0 (0-1) | 5 (3-7) | 210 (80-420) | 360 (195-750) | 81.1 (90) | |
| **Employment status, p**^*^ | **0.000** | **0.000** | 0.468 | 0.432 | **0.195**^†^ | |
| Working full-time hours | 0 (0-1) | 5 (3-7) | 150 (75-280) | 315 (170-520) | 80.7 (276) | |
| Working part-time hours | 0 (0-2) | 5 (3-7) | 175 (100-300) | 330 (175-535) | 79.6 (187) | |
| Not in the labour force | 1 (0-2) | 2 (0-5) | 175 (80-280) | 280 (140-540) | 73.8 (118) | |
| **Student, p**^*^ | **0.000** | **0.000** | 0.832 | 0.887 | 0.885^†^ | |
| Studying full-time | 1 (0 -4) | 2 (1-5) | 150 (80-245) | 300 (174-540) | 78.6 (136) | |
| Not studying full-time | 0 (0-1) | 5 (3-7) | 175 (80-300) | 312.5 (170-525) | 79.1 (451) | |
| **Highest education level**^§^**, p**^‡^ | **0.000** | **0.000** | 0.483 | **0.169** | **0.003**^*^ | |
| Low | 1 (0-4) | 3 (1-6) | 150 (80-245) | 270 (140-535) | 72.9 (110) | |
| Medium | 0 (0 -2) | 5 (2-7) | 175 (75-280) | 330 (140-570) | 74.9 (128) | |
| High | 0 (0 -1) | 5 (3-7) | 175 (80-300) | 330 (180-520) | 82.9 (349) | |
| **Household composition, p**^*^ | **0.072** | **0.030** | **0.057** | **0.029** | 0.216^†^ | |
| Family with children <18yo living at home | 0 (0-1) | 6 (3-7) | 140 (75-210) | 270 (150-460) | 77.3 (160) | |
| Other | 0 (0-2) | 5 (2-7) | 175 (75-300) | 350 (200-540) | 82.0 (196) | |
| **Language spoken at home, p**^*^ | 0.410 | **0.069** | 0.839 | 0.696 | 0.586^\|\|^ | |
| English | 0 (0-2) | 5 (2-7) | 175 (80-280) | 315 (170-530) | 79.0 (571) | |
| Other | 0.5 (0-2) | 3 (0.5-5.5) | 150 (97.5-210) | 250 (150-505) | 80.0 (16) | |
| **General health, p**‡ | 0.231 | 0.225 | **0.001** | **0.000** | **0.000**^*^ | |
| Excellent | 0 (0-1) | 5 (2-7) | 177.5 (100-315) | 422.5 (260-685) | 91.3 (137) | |
| Very good | 0 (0-2) | 5 (2-7) | 180 (90-280) | 330 (200-510) | 83.6 (245) | |
| Good | 0 (0-2) | 5 (2-7) | 162.5 (80-280) | 262.5 (140-495) | 73.9 (161) | |
| Fair/poor | 0 (0-2) | 4 (1-6) | 102.5 (50-210) | 152.5 (60-340) | 53.7 (44) | |
| **Current injury, illness, disability restricting physical activity, p**^*^ | 0.786 | 0.475 | **0.060** | **0.001** | **0.000**^†^ | |
| Yes | 0 (0-1.5) | 5 (2-6) | 125 (47.5-260) | 205 (100-465) | 61.5 (59) | |
| No | 0 (0-2) | 5 (2-7) | 175 (80-280) | 330 (180-540) | 81.6 (528) | |
| **Distance to bus stop, p**‡ | **0.003** | **0.002** | 0.296 | 0.537 | 0.263^*^ | |
| 5 minutes or less | 0 (0-2) | 4 (2-6) | 175 (80-280) | 312.5 (175-515) | 80.1 (383) | |
| 6-10 minutes | 0 (0-3) | 5 (2-7) | 150 (75-350) | 320 (170-545) | 78.3 (126) | |
| 10 or more minutes | 0 (0-0) | 6 (3-7) | 140 (65-260) | 295 (122.5-487.5) | 75.0 (78) | |
| **Access to a motor vehicle, p**^*^ | **0.000** | **0.000** | **0.004** | 0.636 | 0.756^†^ | |
| Access to a motor vehicle | 0 (0-1) | 6 (3-7) | 150 (75-280) | 310 (160-525) | 78.8 (456) | |
| No access to a motor vehicle | 2 (1-5) | 1 (0-2) | 210 (120-315) | 325 (175-540) | 79.9 (131) | |
| **Physical activity last week, p**^*^ | **0.299** | 0.616 | 0.793 | 0.839 | 0.938^†^ | |
| Same as usual | 0 (0-2) | 5 (2-7) | 175 (80-280) | 310 (167.5-527.5) | 79.0 (499) | |
| Different to usual | 0 (0-2) | 54(2-6) | 150 (75-300) | 320 (180-540) | 79.3 (88) | |
| **Urban zone classification, p^*^** | **0.000** | **0.000** | **0.002** | **0.005** | **0.001**^†^ | |
| Inner urban | 0 (0-2) | 3 (2-6) | 180 (100-315) | 350 (210-545) | 85.1 (285) | |
| Middle urban | 0 (0-3) | 5 (2-7) | 140 (70-240) | 282.5 (140-520) | 73.7 (252) | |
| Outer urban | 0 (0-0) | 6 (4-7) | 150 (90-280) | 290 (125-420) | 75.8 (50) | |
| * Kruskal-Wallis test for variables with three or more categories and Mann-Whitney U test for variables with two categories; † Chi-Square test; ‡ Spearman correlation; § Low = Year 12 or less; Medium = Trade/apprenticeship or Certificate/Diploma; High = University qualification; \|\| Fisher’s Exact test | | | | | |  |
